# Chinese Adolescents’ Emotional Intelligence, Perceived Social Support, and Resilience—The Impact of School Type Selection

**DOI:** 10.3389/fpsyg.2019.01299

**Published:** 2019-06-11

**Authors:** Shitao Chen

**Affiliations:** Faculty of Psychology, Beijing Normal University, Beijing, China

**Keywords:** Chinese adolescents, emotional intelligence, perceived social support, resilience, school type

## Abstract

Choosing a school that can best assist children’s development has become one of the major concerns of Chinese parents. To categorize schools by where students stay after school, this paper consider boarding schools or day schools as two major school types. This study examined the relationships among emotional intelligence (EI), perceived social support (from friends and family), and resilience for 493 Chinese adolescents (male = 249, female = 244; mean age = 13.9, SD = 0.71), and investigated how school type difference impacts these relationships. This research first used a moderator analysis to investigate the effects of trait emotional intelligence on resilience by perceived social support from family and friends, respectively. Results show that social support from family was non-significant, while support from friends was significant in moderating the relationship between EI and resilience. Furthermore, a moderated moderation analysis was used to understand if moderation by school type of perceived social support differs in the effect of trait EI on resilience. Results indicated that the magnitude of the moderation by social support from friends depended on school type. For students who have lower perceived friend support, boarding school experiences provided a stronger positive relationship between trait EI and resilience than day school experience. Ways to enhance students’ perceived social support are discussed, along with the limitations of the current research and recommendations for future research.

## Introduction

What makes school choice so important? According to the data published by the Ministry of Education of the People’s Republic of China in 2017, schools have direct contact with 1.4 billion students for at least 6 h a day (MEPRC, 2017). As an important place for children and adolescents to grow, it provides a developmental context, not only for students’ academic learning but also for the growth of their emotional well-being and psychosocial adaptation (Martin and Brown, 2008), their emotional intelligence (Stillman et al., 2018), and their resilience level (Gómez-Baya and Mendoza, 2018). With a proliferation of school choices in China and the intensive educational competition among parents and students, choosing “the best school” for children has become a hot topic (Wu, 2012). Therefore, understanding the factors that might impact students’ development and selecting a school that could best foster students growth is a meaningful decision.

## The Relationships Among Emotional Intelligence, Perceived Social Support, and Resilience

Emotional intelligence (EI) is a popular concept, and the literature on emotional intelligence reveals that this concept is closely connected to one’s academic performance (Sy et al., 2006), quality of social interactions (Summerfeldt et al., 2006; Song et al., 2010), stress management skills (Saklofske et al., 2012), and overall life satisfaction (Palmer et al., 2002). However, the definition of emotional intelligence varies depending upon the theory being used. Cherniss (2010) indicated that there are four dominant theories that are recognized in the field—“Mental Ability” model (Salovey and Mayer, 1989), “Emotional and Social Competence” model (Boyatzis et al., 2000), “Emotional and Social Intelligence” model (Bar-On, 2006), and “Trait Emotional Intelligence Model” (Petrides, 2009). Trait Emotional Intelligence Model is one of the theories that incorporates the key characteristics of the other models and conceptualizes emotional intelligence as an aspect of one’s personality traits (Petrides, 2009).

The Trait Emotional Intelligence Model contains four different large constructs: Emotionality, Self-Control, Sociability, and Well-being. Each construct contains certain facets, with a total of 15 different facets comprising the model. To be specific:

Emotionality is composed of emotion perception, trait empathy, emotion expression, and relationship; Self-Control is composed of emotion regulation, stress management, low-impulsiveness, adaptability, and self-motivation; Sociability is composed of assertiveness, emotion management, social competence, and self-esteem; and Well-being is composed of self-esteem, trait happiness, and trait optimism (Petrides, 2009, p. 95).

Researchers have found that emotional self-awareness, emotional expression, emotional self-control, and emotional self-management appear to be key components in emotional intelligence that serve a central function in psychological resilience (Armstrong et al., 2011), another vital concept that describes one’s ability to successfully cope with challenge or misfortune (Wagnild and Young, 1993).

Resilient individuals are the ones who possess high self-esteem, have strong problem-solving abilities, maintain satisfactory interpersonal relationships, and effectively employ emotion regulation strategies (Tugade and Fredrickson, 2004; Skodol, 2010). The psychological literature has supported the positive relationship between emotional intelligence and resilience (Maulding et al., 2012). For example, Schneider et al. (2013) found that individuals with higher EI would perceive stress as a challenge rather than a threat; they also had less negative affect and showed less distressing psychological response to stress. Another researcher, who targeted a group of high school students (284 girls and 293 boys) found that emotional intelligence is a stronger predictor of resilience compared to cognitive intelligence (Jowkar, 2007).

Recently, resilience research has been a subject of intense interest (Xi et al., 2015). Beyond the finding that emotional intelligence can predict resilience, researchers are now trying to understand what other protective factors can promote one’s resilience. Interestingly, social support, especially social support that is perceived, rather than objectively provided or received (Norris and Kaniasty, 1996), appeared to be a valuable factor in impacting one’s resilience (Wong, 2008). Chan’s (2005) study targeted a group of Hong Kong adolescents and discovered that there is a positive relationship between their perceived social support from family and resilience level. Malecki and Demary (2002) found that although boys and girls in their early adolescence perceive similar levels of support from their parents and teachers, girls perceive more support from classmates and friends. Research results have also revealed that, on one hand, positive peer relationship can predict future school achievement (Zucchetti et al., 2015); on the other hand, adolescents who report having low perceived social support are more likely to experience psychological distress (Klineberg et al., 2006; Jacobson and Newman, 2016), engage in more problem behaviors (Demaray and Malecki, 2002), and have more externalizing and internalizing disorders (Hodges et al., 2016). Other findings showed that adolescents’ adjustment (e.g., school, academic, mental health) is not only predicted by parental support (Rueger et al., 2008) but also impacted by the association between family stress and friendship reciprocity (Ciairano et al., 2007).

Perceived social support is not only related to resilience but also connected with an individual’s emotional intelligence. Petrides et al. (2006) reported that for adolescents who have higher trait EI, they tend to have fewer emotional problems and feel more supported by peers. Poulou (2010) investigated the relationship between personality traits and peer relationships; he reported that individuals who have lower scores on trait EI tend to have more peer problems. This finding is consistent with Petrides et al.’s (2006) report that, for adolescents, regardless of gender, the lower their trait EI, the more behavioral problems they exhibit, which would likely lead to poorer peer relationships. Conversely, students who have high trait EI are supported more by friends and are more likely to be a leader in a group. Gallagher and Vella-Brodrick (2008) further investigated how social support and emotional intelligence predict subjective well-being. Surprisingly, perceived social support was not consistently found to be important for one’s well-being; rather, it was only found to be necessary for people with low emotional intelligence (Gallagher and Vella-Brodrick, 2008). This finding sheds light on the importance of increasing perceived social support for individuals who have lower emotional intelligence.

**Figure 1:**
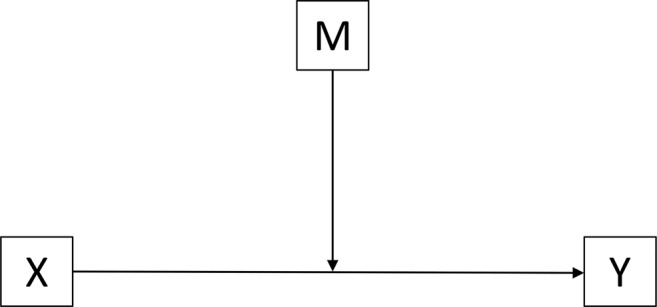
Conceptual model of the relationship among trait EI, perceived social support, and resilience. This model is used to test whether M (perceived social support) moderates the relationship between X (trait EI) and Y (resilience).

Based on the findings above, it appears that perceived social support might be a moderator factor between trait EI and resilience. However, previous research has not examined this specific moderation relationship. Therefore, initially, this study intends to investigate the relationships among these three factors. See Figure 1 for a conceptual model depicting the relationships among trait EI, perceived social support, and resilience.

## School Type Difference and its Impact on Adolescents’ Development

As mentioned above, emotional intelligence, perceived social support, and resilience level are all important factors during adolescent development. However, it is uncertain if school type would make a difference among these variables, since day schools and boarding schools provide different learning and socializing environments for students (Xi, 2011). To be specific, day school focuses on providing regular academic instruction to students, and, as the name implies, students usually go to school during the day and go back home at the end of the school day (Xi, 2011). Even though day schools represent the main form of schooling, boarding schools are also beginning to be a well-established sector of schooling type around the world (Martin et al., 2015). Based on Bronfenbrenner’s (2005) ecological systems theory, boarding school provides a unique socialization environment for students. Students spend most of their time in the same developmental context and they have more opportunities to form personal relationships with teachers, school staff, and peers. This type of interaction is also more regular, stable, and secure (Martin et al., 2014). In addition, boarding school students have more regulations and tight scheduling to follow, their level of school activity involvement is higher than that of day school students, and they tend to form more of a collective identity (Martin et al., 2014).

Though the number of boarding schools is growing and parents are starting to more frequently consider sending their children to boarding school, only a limited amount of research has focused on school type differences and the impact on students’ development. One of the largest studies on boarding school effects on students’ academic and non-academic outcomes indicated that, overall, boarding school students develop more self-discipline and independence than day school students, and they are more mature, better at cooperative learning, and have better critical thinking ability (Martin et al., 2014). Martin and his colleagues also found that students’ academic resilience was higher in a boarding school environment (Martin et al., 2012), boarders who have a non-maladaptive relationship with their parents also tend to develop more personal resources to cope with living away from home (Bramston and Patrick, 2007).

Research does not consistently find positive results for all boarders. Schaverien (2015) described “Boarding School Syndrome” and discussed the trauma experienced by many children who were sent to boarding school at a young age. She found that many boarders adopt unconscious coping strategies to deal with the loss, bereavement and various types of abuse they experienced, which led to a split between the “home self” and the “boarding school self.” This pattern continues into their adult lives and was hypothesized to be the source of considerable emotional distress and relationship difficulties. Though the level of trauma experiences is lower among boarders in recent years, Mander and Lester (2017) corroborated Schaverien’s research, at least in part, with their finding that adolescent boarders reported significantly increased depression, anxiety, emotional symptoms, and hyperactivity over time, compared to nonboarders. Similarly, Behaghel et al. (2017) found that boarders’ levels of well-being were lower when compared to day school students after 1 year. However, their findings revealed that this outcome would be ameliorated after 1 year for students who have the ability to adapt to change. These resilient students tend to benefit more from a boarding school environment when compared to students who have a weaker ability to adjust.

**Figure 2:**
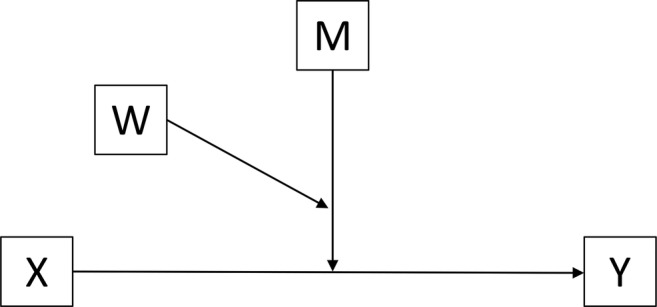
Conceptual model of the relationship among trait EI, perceived social support, resilience, and school type. This model is used to test whether the magnitude of the moderation of M (perceived social support) that works on X (trait EI) and Y (resilience) depends upon W (school type) or not.

Based on the findings above, it appears that boarding school might be an effective environment for some students, but not for others; and various factors, such as an individual’s emotional intelligence (Stillman et al., 2018), perception of social support (Hodges et al., 2016), and ability to recover from adversity (Gómez-Baya and Mendoza, 2018) might all play a role in deciding what type of school would best fit any particular student. Therefore, this research also intends to explore the relationship among these factors to understand how school type selection is impacting Chinese adolescents. Figure 2 demonstrates a conceptual model that adds school type difference as another moderator.

The purpose of this study is, first, to understand the relationships among trait EI, perceived social support from friends and family, and resilience for adolescents. Based on these initial findings, a more important purpose of this study is to understand the role that school type play in impacting the strength of the relationships among the three variables mentioned. Gallagher and Vella-Brodrick (2008) found that perceived social support is important for low trait EI individuals’ well-being, but less so for those with high trait EI. Therefore, it was predicted that school type might impact the level of perceived social support, which, in turn, would influence the magnitude of the relationship between trait EI, perceived social support, and resilience. In summary, understanding these complex relationships provides the opportunity to offer culturally relevant recommendations for parents in their decision-making around the issue of school selection. At the same time, mental health professionals can target a more specific adolescent group when attempting to provide prevention or intervention, so as to enhance this population’s emotional well-being.

## Materials and Methods

### Participants

A total of 507 adolescents from five middle schools (equivalent to grades 7–9 in the U.S. system) in Hangzhou, Zhejiang Province, were recruited for the study. Four hundred and ninety-three students completed the measures. Of the participants who completed the measures, approximately half were male (50.5%, *n* = 249) and half were female (49.5%, *n* = 244). The average age for these students was 13.9, ranging from 13 to 16 (SD = 0.71). A breakdown of participants’ gender, school experience, and age is presented in Supplementary Table S1.

### Procedures

This research project, including all noted attachments, were reviewed and received approval by the Institutional Review Board (IRB) of Indiana University, Bloomington. Prior to recruiting the schools, the researcher sought recommendations from the dean of the local Educational Department in Hangzhou and ultimately selected five middle schools based on students’ heterogeneous backgrounds and whether the schools were representative of regular middle schools in Hangzhou. Among these five schools, two are day schools and have similar school schedules, consistent with what is recommended by the local Education Department. The two boarding schools included in this study also have a similar school environment. The fifth school is a little different from the other schools because it is a mixed type; majority of the students in that school attend the day school section, and students who cannot go back home every day can choose to live at the school. A breakdown of students’ distribution in these schools is presented in Supplementary Table S2. All five schools use the same school curricula, and have around 40 students in each class. Following the school selection, recruiting efforts were made by contacting principals, and, upon receiving permission, classes at each school were randomly selected. After selecting the classes, teachers of these classes were contacted to facilitate the data collection process. Then, student assent forms, parent consent forms and letters to participants were sent. Students could opt out of this study if they chose not to participate; therefore, this was not a passive consent. After obtaining both written informed parental consents and student assents, the questionnaires were distributed by teachers. To increase the confidentiality of the responses, efforts were made to protect students’ privacy; their answer sheets and the questionnaires were returned in sealed envelopes. All students’ answer sheets were read through a preprogrammed computer, and their choices were converted to an excel spreadsheet format.

### Measures

The complete survey was a combination of three different questionnaires, consisting of 69 questions, assessing adolescents’ trait emotional intelligence, perceived social support, and their resilience. Each questionnaire is described below.

#### Trait Emotional Intelligence Questionnaire-Adolescent Short Form-Chinese

Trait Emotional Intelligence Questionnaire-Adolescent Short Form (TEIQue-ASF; Petrides et al., 2006) is a simplified version of the TEIQue, designed specifically for adolescents 12–17 years old. It is composed of 30 statements, rated on a 7-point Likert type scale (1 = *strongly* disagree, 7 = *strongly* agree). Examples of the questions in this scale include: “I am a very motivated person” and “I find it hard to control my feelings” (TEIQue-ASF; Petrides et al., 2006). As with the short version of TEIQue, the TEIQue-ASF was designed to assess adolescents’ global trait EI instead of the factor structures of the construct, because the global score reflects a more holistic picture of one’s trait EI (Petrides, 2010). The TEIQue-ASF has been used with various samples and showed good psychometric properties (Mavroveli et al., 2007). In addition, the global trait EI score obtained from TEIQue-ASF was found to correlate 0.95 with the global score of the long version—TEIQue (Petrides, 2006). The TEIQue-ASF has been translated into over five different languages, including simplified Chinese (London Psychometric Laboratory at UCL, 2015), which was used in this study.

#### Multidimensional Scale of Perceived Social Support

The Multidimensional Scale of Perceived Social Support (MSPSS; Zimet et al., 1988) is a 12-item scale originally designed to measure three sources of support: Family, Friends, and Significant Others. Participants were asked to rate items on a seven-point scale, from 1 = strongly disagree to 7 = strongly agree. Examples of the questions in this scale include: “I get the emotional help and support I need from my family” and “I have friends with whom I can share my joys and sorrows.” The MSPSS has been widely used for participants with diverse ethnic backgrounds and ages and showed adequate psychometric properties (Zimet et al., 1988; Eker and Arkar, 1995; Canty-Mitchell and Zimet, 2000; Chou, 2000; Zhang and Norvilitis, 2002). Zimet et al. (1988) reported good overall internal reliability (Cronbach’s alpha = 0.88) and high subscale internal reliability. The simplified Chinese version of MSPSS was used with an adolescent sample from Hong Kong (Chou, 2000) and Mainland China (Chen, 2017). Both of the studies found that only two factors (Friend support and Family support) were revealed in the adolescent sample. Therefore, this study will only examine perceived social support from these two sources in relation to the other variables discussed in this study.

#### Resilience Scale for Chinese Adolescents

Resilience Scale for Chinese Adolescents (RSCA; Hu and Gan, 2008) is an indigenous scale developed by Chinese scholars. It is a 27-item scale, measuring five different factors: Goal planning, Help-seeking, Family support, Affect control, and Positive thinking. Participants were asked to rate items on a five-point Likert-type scale—1 = completely disagree, and 5 = completely agree. Examples of the questions are, “I am always discouraged by failure”, “Compared to the result, the process is more beneficial to one’s growth”, and “My parents always encourage me to do my best”. Evidence has been provided to indicate adequate psychometric properties, and this measurement was judged to be especially appropriate to assess Chinese adolescents’ resilience (Hu and Gan, 2008; Gan and Yu, 2011; Wen et al., 2015). This scale has been widely used in China in assessing adolescents’ resilience under various situations (Hu and Gan, 2008; Zhou et al., 2011; Wen et al., 2015). Gan and Yu (2011) demonstrated in their study that using the total score of the Resilience Scale derived from the five factors is the best way to measure Chinese adolescents’ resilience level because it captured different facets of resilience. Based upon this finding, the current study will use the total resilience score to represent Chinese adolescents’ resilience level.

### Data Analyses

#### Preliminary Analysis

Before answering the research questions, preliminary analysis of the raw data was conducted. The total missing values constituted less than 3% of the data, and were missing at random (Little’s MCAR test: *χ*^2^ = 22.761, df = 18, *p* = 0.20). For the purpose of obtaining a complete data file, participants with missing data (*n* = 14) were dropped from the sample. Another preliminary analysis was to test the internal consistency of the scales. Cronbach’s alpha (Cronbach, 1951) was used to assess the internal consistency, with results of the reliability tests for all measures presented in Supplementary Table S3. The Cronbach’s alphas ranged from 0.76 to 0.91 and are considered satisfactory.

#### Moderation Analysis

A Moderation Analysis was used to answer the research question regarding whether adolescents’ trait EI’s effect on resilience is contingent on their specific source of perceived social support. The conceptual model is presented in Figure 1. PROCESS is a “computational tool for path analysis-based moderation and mediation analysis as well as their integration in the form of a conditional process model” that was developed by Hayes (2013, p. 419). PROCESS Model = 1 is the best model to fit the path analysis that could address this research question. In this model, predictor variable *X* was the global trait EI value, the outcome variable *Y* was the total score derived from the resilience scale, and the moderator *M* was the subscale score of the perceived social support. To test the moderation relationship, the estimation was that the coefficients of a regression model in which the effect of individual’s trait EI (*X*) on resilience level (*Y*) is allowed to vary linearly with their perceived social support level (*M*) by including the product of *X* and *M* as a predictor of *Y* along with *X* and *M*: *Y* = *i*_1_ + *b*_1_*X* + *b*_2_*M* + *b*_3_*XM* + *e_Y_.*

The key interest of this test was to test *b*_3_, along with an inferential test. If *b*_3_ is not statistically different from zero (*via* a confidence interval test for *b*_3_ that straddles zero), this means that the effect of trait EI is not dependent on perceived social support. But if *b*_3_ is statistically significantly different from zero, it could be concluded that the effect of trait EI on resilience depends on perceived social support.

To ensure the magnitude of the discrepancy in resilience between different trait EI levels was subject to sampling error at each and every value of *M* (perceived social support), a follow up “probing an interaction” test was carried out. Since the perceived social support scale is a quantitative variable, a common strategy when probing an interaction is to use pick-a-point approach. According to Hayes (2013), this approach could “estimate the conditional effect of *X* on *Y* when *M* is equal to mean, a standard deviation below the mean, and a standard deviation above the mean” (p. 236). However, since it is arbitrary to use plus and minus one standard deviation from the mean to represent “low,” “moderate,” and “high” on the moderator, the Johnson-Neyman (JN) technique was applied as well. The JN technique might generate a single solution within the range of the measurement of the moderator to indicate a statistically significant transition point in the observed moderators. It is also possible to have no solution within the range of the moderators. This could either mean the conditional effect of *X* on *Y* was statistically significant across the entire range of the moderator, or the conditional effect of *X* on *Y* was not statistically significant anywhere in the observed distribution of the moderator.

#### Advanced Moderated Analysis

Moderated moderation model is an advanced moderation model that was used to answer the second research question—whether the magnitude of the moderation by perceived social support of the effect of trait EI on adolescents’ resilience depends upon school type (W). Two identical moderated moderation models were used to assess the three-way interaction among trait EI, perceived social support (from family and friends respectively), and school type. A conceptual model of this analysis is presented in Figure 2.

PROCESS, model 3 is built to simplify the estimation of a moderated moderation model. Model 3 identified the outcome variable Y (resilience), focal predictor X (trait EI), the primary moderator M (perceived social support), and secondary moderator W (school type). PROCESS calculated all the necessary products, estimated the best-fitting ordinary least square (OLS) regression model, and probed the interaction. For probing the interaction, the pick-a-point approach was used to select the value of perceived social support on its low, moderate, and high level (mean and plus/minus one SD from mean), and assessed whether school type moderates trait EI’s effect on resilience, conditioned on these selected values of perceived social support. The Johnson-Neyman (JN) technique was applied here as well to generate a single solution within the range of the measurement of the moderator to indicate a statistically significant transition point in the observed moderators.

## Results

### Moderation Analysis

**Figure 3:**
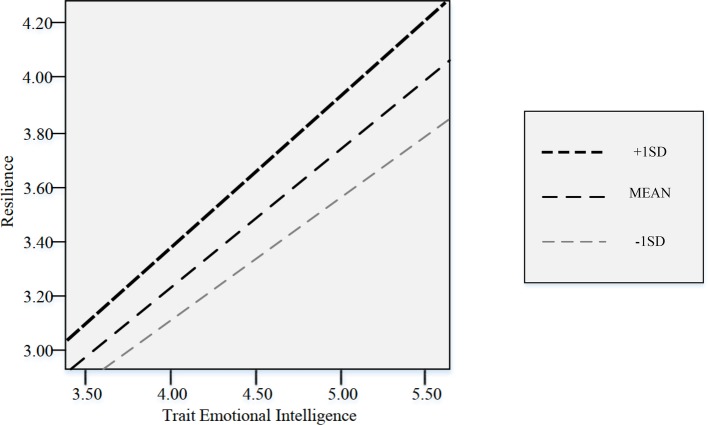
Results of the moderation model. Three lines are the visual representation of different moderation effects of Trait EI on resilience when perceived social support from friends scores were at its +1 SD, mean, and −1 SD.

To investigate the effects of trait Emotional Intelligence on resilience by perceived social support from Family and Friends, respectively, two identical moderation analyses were conducted, using PROCESS model 1 in SPSS (version 22). In the first analysis, the interaction between trait EI and perceived social support from Family (*b*_3_) was not statistically significant, *t*(489) = 0.56, *p* > 0.05, 95% CI (−0.02; 0.04). Based on data from the current sample, then, Chinese adolescents’ trait EI’s impact on resilience was not moderated by their perceived social support from family. In the second analysis, the interaction between trait EI and perceived social support from friends emerged as a significant predictor, *t*(489) = 2.31, *p* < 0.05, 95% CI (0.01; 0.06), *R*^2^ = 0.60, *F*(3,489) = 243.04, *p* < 0.001 (Supplementary Table S3); and the *R*^2^ had a statistically significant change due to the interactions, *F*(1,489) = 5.32, *p* = 0.022, changing *R*^2^ = 0.0044. This means the moderation component of the model explained about 60% of the variance in resilience. The result indicated that the effect of adolescents’ trait EI on their resilience level depends on their perceived social support from friends. Figure 3 illustrates the interaction by depicting the regression lines of the relationship between trait EI and resilience at high, medium and low (+1 SD, mean, −1 SD) scores of the subscale score of MSPSS Friends. The fan pattern of the figure showed that Perceived Social Support from Friends functioned as a magnifier for the positive relationship noted in the research literature between adolescents’ trait EI level and their resilience. The highest level of resilience occurred in individuals who reported high trait EI and high perceived social support from friends. Overall, then, the moderation analyses results indicated that perceived social support from friends, but not from family, enhanced the relationship between Chinese adolescents’ trait Emotional Intelligence and resilience.

### Advanced Moderation Analysis

To understand if the moderation by gender of perceived social support differs in the effect of trait EI on resilience, two moderated moderation analyses were conducted through PROCESS model 3 on SPSS (version 22). The regression coefficients for both of the three-way interactions were not statistically significant [when perceived social support from family was employed as one of the moderators, *t*(485) = 0.22, *p* = 0.82; and when perceived social support from friends was used as one of the moderators, *t*(485) = −1.48, *p* = 0.14]. This means the magnitude of the moderation by perceived social support of trait EI on resilience level did not depend on gender.

When investigating if the moderation by school type of perceived social support differs in the effect of trait EI on resilience, there was no evidence of a three-way interaction among trait EI, perceived social support from family, and school type on resilience level, *t*(485) = −1.10, *p* = 0.27. However, there was a statistically significant interaction among trait EI, perceived social support from friends, and school type on resilience level, *t*(485) = −2.19, *R*^2^ increase = 0.004, *F*(1,485) = 4.78, *p* = 0.03 (Supplementary Table S4). This means that the magnitude of the moderation by perceived social support from friends on trait EI depended closely on school type, which impacted adolescents’ resilience level.

**Figure 4:**
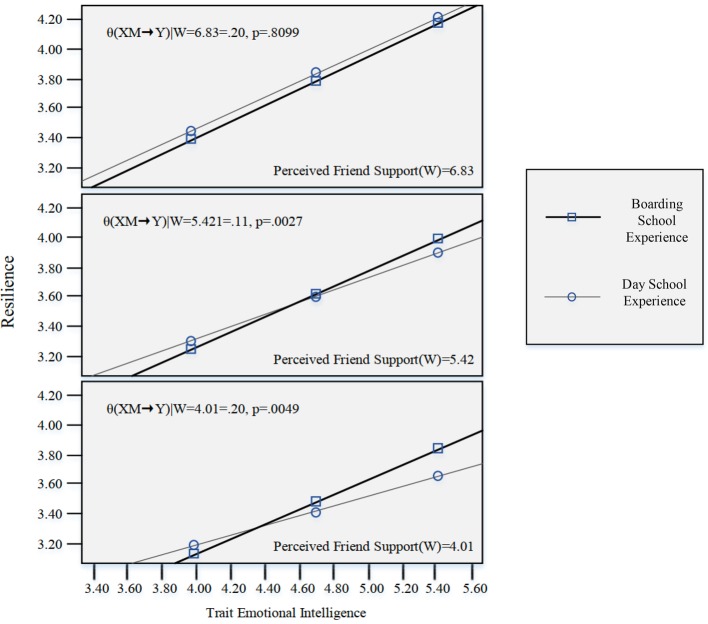
Results of the moderated moderation model. This is a visual representation of the moderated moderation analysis, examining the moderation by school type of perceived social support from friends’ differences in the effect of trait EI on resilience.

When probing the interaction using the pick-a-point approach, the visual representation of this model (Figure 4) shows that the effect of trait EI on resilience was consistently positive, and there was a statistically significant school type difference when perceived social support was at a low [*θ*_(*XM*→*Y*)|*W* = 4.01_ = 0.20, *p* = 0.0049] to moderate level [*θ*_(*XM*→*Y*)|*W* = 5.42_ = 0.11, *p* = 0.0027]. However, when perceived social support was at a high level [*θ*_(*XM*→*Y*)|*W* = 6.83_ = 0.02, *p* = 0.8099], the school type difference was not statistically significant. To be more specific, there was a statistically significant difference in the effect of trait EI on resilience between students with boarding school experience and day school experience among those whose perceived social support from friends score was less than 5.46. Above this score, school experience did not moderate the effect of trait EI on resilience. Practically speaking, when perceived social support from friends was in the low to moderate range, trait EI was more strongly associated with resilience among boarding school students than among day school students. This result partially validated the hypothesis that school type would impact the relationship between trait EI, perceived social support, and resilience. This finding also has practical implications for school selection, as well as clinical implications, providing a rationale for targeting students with lower social support from friends.

## Discussion

The results of this study indicate that perceived social support from friends, but not from family, served a moderating role between trait EI and resilience. In addition, for adolescents whose perceived social support from friends was at a low to moderate level, there was a statistically significant difference in the effect of trait EI on resilience level between students with boarding school experience and those with day school experience. The reasons for these results will be discussed in a subsequent section of the paper.

### The Moderator Role of Perceived Friend Support for Adolescents

Perceived social support serves an important role in people’s lives, especially for adolescents, whose developmental goals include, but are not limited to, becoming independent from parents, establishing closer connections with a peer group, forming close friendships, and developing a romantic relationship (Pfeiffer et al., 2016). Consistent with this, the findings that emerged from this study demonstrate that perceived social support from friends is more influential than family for adolescents at this stage of their development. To be specific, for adolescents who have similar levels of trait EI, the higher the friend support they perceived, the more resilient they were in facing adversity and challenge. The powerful influence of perceived friend support replicated what had been found previously. Specifically, friend support exceeded the importance of parent support for students in late adolescence (Bokhorst et al., 2010), and friendship was rated as the most important source of social support among a group of Asian immigrant adolescents (Thomas and Baek Choi, 2006). The reasons behind the weighty role perceived friend support played in this study might be related to adolescents’ developmental need of having a sense of intimacy with and support from friends (Furman and Buhrmester, 1992). The great intimacy among peers can satisfy their need to be recognized socially (Collins and Laursen, 2004), and feeling supported by their peers, especially reciprocal friendship, can give them a sense of belonging, which, in turn, increases their courage to face adversity and decrease their aggressive behavior towards challenges (Ciairano et al., 2007). However, this finding does not mean that perceived social support from family is not important. Parental support has been shown to play a critical role in children’s development and serves as a foundation for their sense of secure attachment (Bowlby, 1969/1982). Research also supports the idea that when children have better attachment and perceived high social support from parents when they are young, they are more likely to perceive and seek peer support in their adolescence (Szwedo et al., 2017). Moreover, when family creates a high stress environment, supportive friendship appeared not to have a positive impact on adolescents’ expectation for success and sense of belonging (Ciairano et al., 2007). Therefore, supportive parents and positive family environment, even though secondary in importance to peer support during adolescence, continues to be an important factor that should not be ignored.

Since the current study reveals that the higher the perceived friend support, the stronger the positive relationship between trait EI and resilience, it is vital to increase adolescents’ awareness of social support from their social network, especially from their peers. To increase one’s perceived social support, utilizing gratitude practice has been found to be one evidence-based strategy (Wood et al., 2008). In related fashion, keeping gratitude journals and reflecting upon simple moments that one perceives as a gift (Emmons and McCullough, 2003) can also increase one’s perceived social support. It is recommended that mental health professionals, teachers, and parents intentionally provide opportunities for adolescents to try these strategies so as to enhance their ability to feel supported by others.

### School Type Selection Impact on Adolescents’ Development

Another result from the current study reveals that, for students who have lower perceived friend support, boarding school experience (e.g., attending boarding school or mixed type school with staying at school during the week as an option) was a better choice for those who had high trait EI (total trait EI score equal to or greater than 4.5). To understand this finding, students’ trait emotional intelligence and school culture need to be evaluated together. First, students with higher trait EI are more likely to have stronger ability to regulate their emotions and manage their relationships with others (Zolkoski and Bullock, 2012). Though some of these students do not perceive high friend support, they can still manage their interpersonal relationships well, fit into the boarding school environment, and seek additional support. Second, school and teacher support are important protective factors in a student’s life. While Chinese day school teachers devote most of their attention on students’ academic performance (Zhang et al., 2013), boarding school teachers are involved in students’ daily lives, in addition to their regular role, and offer more resources to students. Thus, boarding school students spend more quality time with supportive teachers and school personnel (Tian et al., 2013). This explained the higher resilience level that boarding school students with high trait EI presented, compared to day school students, even though their perceived friend support was at the low to medium level (Zolkoski and Bullock, 2012).

Nevertheless, for students who obtained scores indicative of a similar level of perceived friend support (lower than 5.46), but with lower trait EI, day school was a better choice. These students were more likely to feel overwhelmed in boarding school environments that promote independence, autonomy, and assertiveness; some of them might even be bullied or experience other relational victimization (Pfeiffer and Pinquart, 2014). Due to their inadequate social skills and their higher levels of emotionality, for these students, a boarding school’s environment becomes a risk factor that might potentially decrease adolescents’ sense of school belongingness and increase their feelings of loneliness. On the contrary, day school offers a contained and time-limited space for students to focus on academics in school and recover after school, which makes day school a better choice that can slightly increase their resilience.

### Implications

As the first study to explore the role school type played among trait EI, perceived social support, and resilience, it has implications for parents and mental health professionals. For parents, this study provides some guidance on school selection for their children, which includes considering their emotional intelligence level and their perceived friend support as two important factors. In general, adolescents with high perceived friend support would fit in any type of school and have a high resilience level. However, when perceived friend support is lower, adolescents with higher trait EI are more resilient in a boarding school environment, while adolescents with lower trait EI fit better in day school environment. This result validates Qualter and his colleagues’ finding that students with average to high EI cope better with school transition than students with low EI (Qualter et al., 2007). Therefore, parents should evaluate adolescents’ trait EI level and their perception of the friend support they would likely receive in school before making this school choice decision, so that they can create an optimal fit in the school environment and enhance their psychological well-being. Nevertheless, if boarding school is a choice that has already been made, and adolescents were having some adjustment difficulties and revealing psychological distress, parents can consider helping adolescents to learn some emotional intelligence strategies, so that they can improve their skills in facing these challenges (Qualter et al., 2007).

Furthermore, school professionals play a significant role in providing prevention and intervention to cultivate students’ psychological development (Van Ryzin, 2011). One of the fundamental differences between boarding school and day school experience is the level of emotional support and quality time school professionals provide to students. School professionals, no matter what school setting they are in, are recommended to form positive relationships with students, be good role models in their lives, and enhance adolescents’ feelings of worthiness and belonging. They can also encourage students to write gratitude journals and keep regular contact with parents and friends in order to foster a smooth adaptation to the new environment. For students who are lacking adequate abilities in emotion regulation, stress management, and social interaction, school mental health professionals can provide individual counseling or group counseling services to teach students skills that would allow them to be more emotionally intelligent and to be more prepared for the inevitable difficult times.

### Limitations and Future Directions

Beyond the implications, there are some areas needing improvement in future studies. First, only two types of social support (from friends or family) were examined in this study, and the data were all collected through a self-report format. Due to the vital role that teacher support and support from other social resources play in an adolescents’ life (Van Ryzin, 2011), future studies should at least consider using measures that include perceived social support from teacher as an important variable. Future studies should also collect data that extends beyond the self-report format. Parents and teachers’ feedback regarding students’ emotional intelligence and resilience, and support they provide to students, might be interesting moderators to examine. Second, this study included adolescents from 13 to 16 years old (mean = 13.9, SD = 0.71), an age period that did not well represent the entire adolescent stage. Since adolescents experience dramatic changes in terms of their emotion regulation abilities, their ability to solve problems or establish relationships, there might be a different trend between preadolescents, early adolescents, and late adolescents. Future studies can therefore consider making a comparison between different adolescent age groups. Third, even though the five schools selected in this study may represent normal middle schools in Hangzhou, each school and even the randomly selected classes have their unique culture. For instance, some schools or classes have a more liberal environment than the others, or some have formed a more supportive and collaborative classroom environment. Since these subcultures were not examined in this study, it can be an important piece that impacts adolescents’ development. Last but not least, the cultural diversity within China requires special considerations regarding of the generalizability of the research results. Hangzhou is the fourth-largest metropolitan area in China and attracts people from all over the country. Population demographics in Hangzhou are much more diverse than those from the western or more rural areas of China. Therefore, the findings and conclusions of this study may only be applicable to students from eastern, metropolitan areas of China. To form a well-rounded understanding of the connection between Chinese adolescents’ trait EI, perceived social support, resilience, and school type, future research can replicate this study, but increase generalizability by selecting adolescents with a broader age range from western and less-developed parts of China. In addition, future studies can consider collecting information regarding students’ family socioeconomic status, parents’ educational level, and early parent-child attachment style as variables controlled before analyzing data, in order to better understand the complex interactions among adolescents’ trait EI, perceived social support, and resilience.

## Ethics Statement

This study was carried out in accordance with the recommendations of Institutional Review Board (IRB) of Indiana University Bloomington, with written informed consent from all subjects. All subjects gave written informed consent in accordance with the Declaration of Helsinki. The protocol was approved by the Institutional Review Board (IRB) of Indiana University Bloomington.

## Author Contributions

SC is the sole author who initiated this study, collected data, ran the data analysis, and completed the manuscript.

### Conflict of Interest Statement

The author declares that the research was conducted in the absence of any commercial or financial relationships that could be construed as a potential conflict of interest.
