# Supplementary material for: Chinese Adolescents’ Emotional Intelligence, Perceived Social Support, and Resilience—The Impact of School Type Selection
Source: Front Psychol. 2019 Jun 11;10:1299. doi: 10.3389/fpsyg.2019.01299 (PMC6579893; doi:10.3389/fpsyg.2019.01299)
Supplement: Supplementary file 1 [file Table_1.DOCX]

Supplementary Material

Table 1

*Break Down of Participants’ Age Distribution by School Type and Gender.*

|  | Day School | | Boarding School | | Total | |
| --- | --- | --- | --- | --- | --- | --- |
|  | n | M(SD) | n | M(SD) | n | M(SD) |
| Males | 127 | 13.9(.76) | 122 | 13.8(.68) | 249 | 13.9(.72) |
| Females | 111 | 13.9(.75) | 133 | 13.9(.67) | 244 | 13.9(.71) |

Table 2

*Break Down of the Student Distribution by 5 Different Schools*

|  | Day School | | |  | Boarding School | | |
| --- | --- | --- | --- | --- | --- | --- | --- |
| Gender | School 1 | School 2 | School 3 |  | School 1 | School 4 | School 5 |
| Male | 31 | 38 | 58 |  | 9 | 58 | 55 |
| Female | 26 | 44 | 41 |  | 9 | 62 | 62 |
| Total | 57 | 82 | 99 |  | 18 | 120 | 117 |

*Note*. School 1 is a mixed type school, therefore students who were randomly selected to participate this study might have different school type experience.

Table 3

*Reliability Test Results for TEIQue-ASF, MSPSS Subscales, and RSCA.*

| Measures | Number of Items | Cronbach’s Alpha |
| --- | --- | --- |
| TEIQue-ASF | 30 | 0.82 |
| MSPSS-Friends | 8 | 0.91 |
| MSPSS-Family | 4 | 0.76 |
| RSCA | 27 | 0.88 |

Table 4

*Results from a Regression Analysis Examining the Moderation of the Effect of trait Emotional Intelligence on Resilience level by Perceived Social Support from Friends*

|  |  | Coeff. | SE | *t* | *p* | 95% CI |
| --- | --- | --- | --- | --- | --- | --- |
| Intercept | i_1_ | 1.43 | .38 | 3.80 | .0002 | [.69 , 2.17] |
| Trait Emotional Intelligence (X) | b_1_ | .32 | .09 | 3.78 | .0002 | [.16 , .49] |
| Perceived Social Support Friends (M) | b_2_ | -.04 | .07 | -.63 | .53 | [-.18 , .09] |
| Trait Emotional Intelligence * PPS Friends (XM) | b_3_ | .03 | .02 | 2.31 | .022* | [.01 , .06] |
|  |  | *R^2^*=.60, MSE=.15 | | | |  |
|  |  | *F*(3,489)=243.04, *p* < .001 | | | |  |

*Note.* CI=confidence interval.

*p<.05.
